# Application and usefulness of a new eight‐wire basket catheter for endoscopic extraction of small common bile duct stones: A retrospective multicenter study

**DOI:** 10.1002/deo2.138

**Published:** 2022-06-05

**Authors:** Osamu Inatomi, Masanobu Katayama, Koichi Soga, Takashi Yamamoto, Takao Mikami, Yukihiro Morita, Jun Nakajima, Shuhei Shintani, Yuki Yagi, Yuki Kishi, Kazuyoshi Matsumura

**Affiliations:** ^1^ Department of Medicine Shiga University of Medical Science Shiga Japan; ^2^ Department of Gastroenterology Saiseikai Shiga Hospital Shiga Japan; ^3^ Department of Gastroenterology Omihachiman Community Medical Center Shiga Japan; ^4^ Department of Gastroenterology Koka Public Hospital Shiga Japan; ^5^ Department of Gastroenterology Otsu Red Cross Hospital Shiga Japan; ^6^ Department of Gastroenterology Hikone Municipal Hospital Shiga Japan; ^7^ Department of Gastroenterology Otsu Municipal Hospital Shiga Japan; ^8^ Department of Gastroenterology Shiga General Hospital Shiga Japan

**Keywords:** biliary stones, common bile duct stones, eight‐wire basket catheter, endoscopic retrograde cholangiopancreatography, stone extraction

## Abstract

**Objectives:**

Distally located small common bile duct stones are often difficult to treat or grasp endoscopically. Therefore, multiple devices, such as baskets or balloon catheters, are frequently used in such cases. However, it is desirable to use a single device for stone extraction from the perspective of cost‐effectiveness. In this multicenter study, we evaluated the efficacy of a new eight‐wire basket catheter for extracting small (≤10 mm) common bile duct stones.

**Methods:**

We retrospectively analyzed the records of 144 patients who underwent stone extraction using the eight‐wire basket catheter for common bile duct stones ≤10 mm. The success rate of complete stone extraction and the risk factors for the difficulty in stone extraction with the eight‐wire catheter alone were mainly evaluated.

**Results:**

The success rate of stone extraction with the eight‐wire catheter alone was 86.1%. The final rate of complete stone extraction was 98.0%. The mean of the maximum diameter of the common bile duct and the largest stone dimension were 10.5 ± 3.5, and 5.1 ± 2.1 mm, respectively. Common bile duct diameter ≥12 mm and stone diameter ≥6 mm were identified as independent risk factors for the difficulty in stone extraction with the eight‐wire catheter alone.

**Conclusions:**

The success rate of the new eight‐wire basket for small common bile duct stone extraction was acceptable. The device is beneficial and could be used from the start for the extraction of small stones < 6 mm.

## INTRODUCTION

Bile duct stones are a major cause of acute cholangitis that may end fatally due to sepsis. Therefore, therapeutic intervention is necessary even if the patient is asymptomatic.^1^, ^2^ Generally, large, or piled up stones are the most difficult to be endoscopically treated. Moreover, small stones ≤10 mm are sometimes difficult to be extracted as they may be stuck in a pocket‐like region at the lower end of the distal common bile duct.^3^, ^4^

According to previous reports, eight‐ or six‐wire baskets and balloon catheters are the most suitable devices for extracting such small stones. Stone extraction using the basket catheter may be difficult in a single session when the number of stones is large, and the balloon catheter may allow stones to slip through when the stone diameter is small.^5^, ^6^ In such cases, multiple devices are often used, but it is preferable to use a single device in one session for stone extraction to increase the cost‐effectiveness of the procedure. However, the therapeutic outcomes of different devices for the removal of small stones have not been fully investigated.

The new eight‐wire basket catheter made of nitinol has a more flexible structure, which can be easily deformed to fit the shape of bile ducts compared with the conventional stainless‐steel catheters. In this retrospective multicenter study, we investigated the efficacy of the new eight‐wire basket catheter for treating small (≤10 mm) common bile duct stones.

## METHODS

### Patients

**FIGURE 1:**
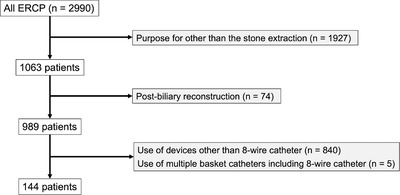
Flowchart of the eligibility criteria of the study

Among 2990 patients who underwent endoscopic retrograde cholangiopancreatography (ERCP) from January 2020 to March 2021 at eight facilities in Shiga Prefecture in Japan, 144 patients underwent stone extraction using an eight‐wire basket catheter (Medi‐Globe 8‐Wire Nitinol Basket; Medico's Hirata Inc, Osaka) as the first‐line device for common bile duct stones ≤ 10 mm. Patients with post biliary reconstruction and those in whom multiple basket catheters were used for stone extraction were excluded from the study (Figure 1 shows the eligibility criteria for the study). The presence of stones or residual stones was assessed via ERCP‐based cholangiography during the ERCP procedure after stone extraction. All ERCP‐related data were stored as an electronic database and a video at each institute. Informed written consent for ERCP was already previously obtained from each patient as a routine before the procedure, and informed consent for the study was obtained in an opt‐out form on the website. This study was approved by the ethics committee of the Shiga University of Medical Science (No. R2021‐038) and conducted in accordance with the Helsinki standards, 2013.

### Eight‐wire nitinol basket catheter

**FIGURE 2:**
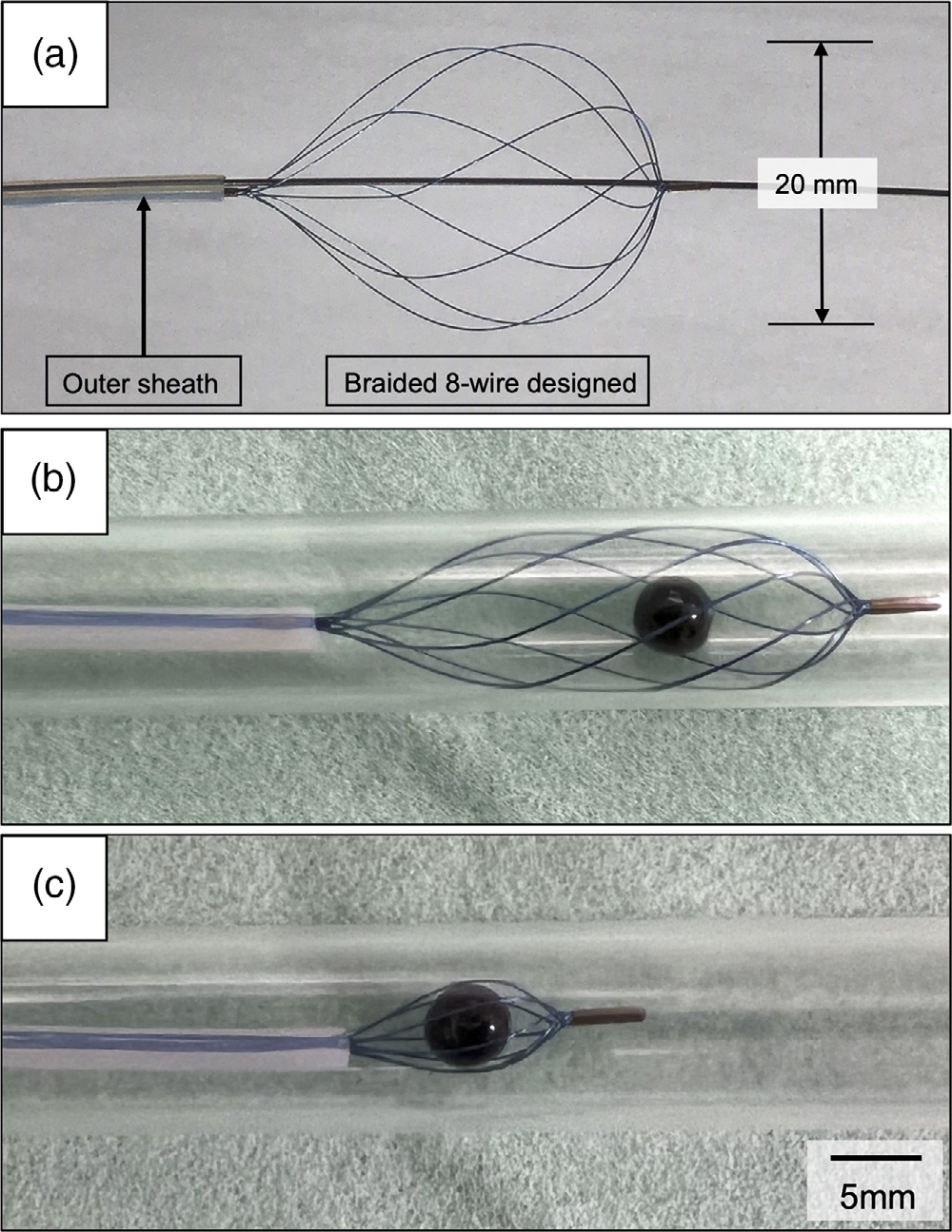
(a) The structure of the eight‐wire basket catheter. The basket portion consists of eight wires made of nitinol. The guidewire is directed coaxially with the tip of the basket. The outer sheath is made of plastic with a diameter of 8.5‐Fr. (b) An eight‐wire basket catheter deployed in a glass tube with a 10‐mm lumen and a bead that resembles a 5‐mm diameter stone. The shape of the basket portion is formed to follow the shape of the lumen. (c) The 5‐mm diameter bead can be easily grasped.

The catheter consists of eight wires made of nitinol in the basket portion at the tip. The basket can be rotated by manipulating the handle of the device. It is double‐lumened, guidewire‐guided, with a plastic sheath of 8.5‐Fr and a maximum diameter of 20 mm when deployed. In a mockup experiment, we evaluated the deformation of the basket catheter when it was deployed, and the beads were grasped using a glass tube with 10–mm diameter lumen and 5–mm beads to simulate bile ducts and stones. (Figure 2)

### Endoscopic procedures

A side‐viewing duodenoscope JF260V or TJF290V (Olympus Optical Co., Tokyo, Japan) and guidewire Visiglide2 (Olympus) were used for ERCP conduction. A combination of midazolam and pentazocine or dexmedetomidine or propofol was used for conscious sedation. Stones of ≤10 mm were confirmed via cholangiography or intraductal ultrasound, and the eight‐wire basket was inserted as the first device for stone extraction. When multiple attempts with the basket catheter failed to extract stones, a balloon catheter (Multi‐3V Plus; Olympus, X‐TraX Multi‐Stage; Medico's Hirata, Extracto Pro; Boston Scientific, Marlborough, Mass, USA, Extraction balloon catheter; Zeon Medical, Tokyo, Japan) was added. The clearance of all present stones was confirmed via ERCP‐based cholangiography during the ERCP procedure after stone extraction, and follow‐up to evaluate the recurrence of the stones was not routinely performed in this study. The decision to use endoscopic sphincterotomy (EST), with endoscopic papillary balloon dilation (EPBD) or only the EPBD was made at the discretion of the surgeon.

### Outcomes

The primary endpoint was the success rate of complete stone extraction with the eight‐wire basket catheter alone, and the secondary endpoints were the risk factors for the difficulty in stone extraction with the eight‐wire catheter alone, the frequency of additional use of balloon catheters for stone extraction, the rate of complete stone extraction, whether a trainee was involved, maximum bile duct diameter, stone diameter, number of stones, number of basket catheter sweeps, complication rate, and procedure time for stone extraction (time from reaching the papilla to the end of the endoscopic procedure). A trainee is an endoscopist who had performed ERCP procedures within 5 years.

### Statistical analysis

Student's t‐test or the Wilcoxon rank‐sum test were used for comparison of continuous variables pertaining to the baseline characteristics of the two groups as appropriate and chi‐square or Fisher's exact test was used for categorical variables. A *p*‐value of < 0.05 was considered statistically significant. For the difficulty in stone extraction with the eight‐wire catheter, cutoff values for maximum common bile duct diameter and maximum stone diameter were calculated using receiver operating characteristic (ROC) analysis. Multivariate logistic regression analysis was performed for maximum bile duct diameter, maximum stone diameter, and the number of stones more than or equal to four as risk factors for stone extraction difficulty. All analyses were performed using SPSS version 25.0 (SPSS Inc., Chicago, IL, USA) and Prism version 9.0 (GraphPad, San Diego, Calif.).

## RESULTS

For the 144 patients, the mean age was 73.5 ± 13.8 years, the mean common bile duct diameter was 10.5 ± 3.5 mm, the mean number of stones was 2.4 ± 2.2, and the mean stone diameter was 5.1 ± 2.1 mm (Table 1). The success rate of complete stone removal with the eight‐wire basket catheter alone was 86.1%. A balloon catheter for stone extraction was used additionally after the use of the basket catheter in 18 (12.5%) patients. The mean procedure time for stone extraction was 18.4 ± 10.9 min. Complications occurred in 2.7%, in the form of post‐ERCP pancreatitis in two patients and post‐EST bleeding in two patients. The severity of the complications was mild in all cases. By using an additional balloon catheter, the final complete rate of complete stone extraction was 98.0% (Table 2). There were two cases in which stones could not be extracted with the basket alone, and the procedure was terminated without the addition of other devices due to patient‐related conditions.

**TABLE 1:** Patient characteristics

	***n* = 144**
Age, year (mean ± SD)	73.5 ± 13.8
Sex, male, *n* (%)	89 (61.8)
ASA ≥ 3, *n* (%)	13 (9.0)
Acute cholangitis, *n* (%)	43 (29.9)
Naïve papilla, *n* (%)	90 (62.5)
Duodenum diverticulum, *n* (%)	40 (27.8)
Common bile duct diameter, mm (mean ± SD)	10.5 ± 3.5
Number of stones, *n* (mean ± SD)	2.4 ± 2.2
Diameter of the largest stone, mm (mean ± SD)	5.1 ± 2.1

SD; standard deviation.

ASA; American Society of Anesthesia Classification (Owens *et al*. *Anesthesiology* 1978; **49**: 239–43.).

**TABLE 2:** Outcomes and complications

	***n* = 144**
Trainee, *n* (%)	58 (40.3)
Procedures, *n* (%)	
EST	115 (79.8)
EPBD	23 (16.0)
Procedure time*, min (mean ± SD)	18.4 ± 10.9
Number of the basket catheter sweep, *n* (mean ± SD)	2.7 ± 1.5
Stone extraction by the basket catheter alone, *n* (%)	124 (86.1)
Additional use of balloon catheter, *n* (%)	18 (12.5)
Complete stone extraction, *n* (%)	142 (98.0)
Complications, *n* (%)	
PEP	2 (1.3)
Bleeding	2 (1.3)

Abbreviations: EPBD, endoscopic papillary balloon dilation; EST, endoscopic sphincterotomy; PEP, post‐endoscopic retrograde cholangiopancreatography pancreatitis.

* From reaching the papilla to the end of the endoscopic procedure.

Table 3 shows the results of regression analyses (univariate and multivariate) of risk factors for the difficulty in stone extraction using the eight‐wire basket catheter alone. In univariate analysis, the presence or absence of acute cholangitis before ERCP, and duodenum diverticulum did not represent risk factors for difficult stone extraction by the eight‐wire basket. The multivariate analysis indicated that the diameter of the stone (OR 2.9, 95% CI 1.0–8.3, *p* = 0.04), the maximum diameter of the common bile duct (OR 3.1, 95% CI 1.1–8.7, *p* = 0.03) were independent risk factors for difficult extraction.

**TABLE 3:** Univariate and multivariate analysis of risk factors for failed stone removal with the eight‐wire basket catheter alone

	**Univariate analysis**		**Multivariate analysis**	
**Parameters**	**OR (95% CI)**	***P***	**OR (95% CI)**	***P***
Sex, male	1.8 (0.7–4.6)	0.24		
Age	1.8 (0.7–4.5)	0.24		
Duodenum diverticulum	1.1 (0.4–3.2)	0.81		
Acute cholangitis before ERCP	1.3 (0.5–3.6)	0.59		
Trainee	1.8 (0.7–4.6)	0.23		
EST	1.9 (0.7–5.3)	0.24		
EPBD	0.5 (0.2–1.5)	0.32		
Number of stones	1.1 (0.9–1.2)	0.56		
Diameter of the largest stone	3.3 (1.2–8.7)	0.02	2.9 (1.0−8.3)	0.04
Maximum diameter of common bile duct	4.6 (1.5−13.6)	< 0.01	3.1 (1.1−8.7)	0.03

Abbreviations: EPBD, endoscopic papillary balloon dilation; ERCP, endoscopic retrograde cholangiopancreatography; EST, endoscopic sphincterotomy.

**FIGURE 3:**
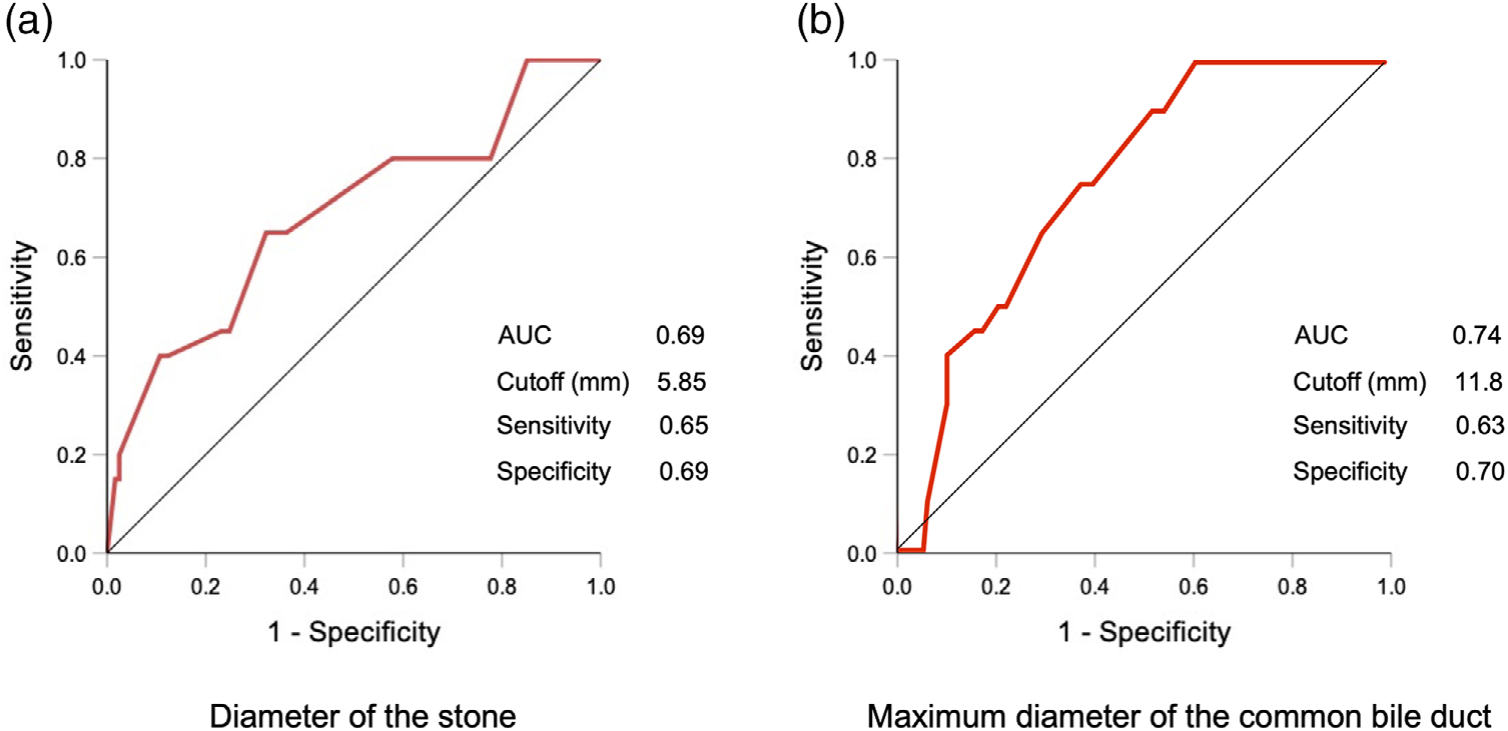
Receiver operating characteristic curves of (a) the diameter of the stone and (b) a maximum diameter of the common bile duct for the difficulty of extraction using the eight‐wire basket catheter alone

In ROC analysis, when using 5.9 mm as a cutoff value for the diameter of the largest stone, its sensitivity for diagnosing difficult stone extraction was 65.4%, specificity was 69.3%; and area under the curve (AUC) was 0.69 when using 11.8 mm as the diameter of the common bile duct, the sensitivity was 63.2%, specificity was 69.7%, and AUC was 0.74 (Figure 3). Comparisons between the success and failure groups in stone extraction with the eight‐wire catheter alone per each risk factor are shown in Table S1.

## DISCUSSION

This study demonstrated the usefulness of the novel eight‐wire basket catheter for extracting small common bile duct stones (≤ 10 mm). In most cases, stone extraction was completed without a balloon catheter. Further, the eight‐wire basket catheter was effective in the extraction of multiple common bile duct stones (four or more stones) and for small (< 6 mm) stones, which had been considered difficult to extract in previous reports.^6^

Recent advances in endoscopic devices for common bile duct stone extraction have been significant.^4^, ^7^, ^8^ However, there is no ideal effective device for all types of stones, and therapeutic strategy depends on the availability of devices, and the associated circumstances.^9^, ^10^, ^11^, ^12^, ^13^, ^14^

There is still debate regarding whether basket or balloon catheters are more effective for extracting stones < 10 mm. Ishiwatari et al. reported in a randomized controlled trial that the success rate of stone extraction is 92.3% with balloon catheters and 80% with basket catheters (eight‐wire in the tip and four‐wire in the proximal structure) in patients with stone diameters ≤10 and bile duct diameters ≤15 mm. In addition, they reported that the balloon catheter was significantly superior for the extraction of multiple stones (≥4).^5^ Ozawa et al. reported that the success rate of stone extraction for bile duct stones < 11 mm in diameter was comparable in both balloon catheters and four‐wire basket catheters.^6^ They found that the success rate of stone extraction within 10 min was 83.9% for balloon catheters (retrieval balloon, B‐V232/242/432/442; Olympus) and 81.3% for basket catheters (four‐wire retrieval basket, FG‐V436P; Olympus). Furthermore, their study reported that stones < 6 mm were independent risk factors for difficulty in stone extraction. They observed that the stones, which were difficult to retrieve were embedded into a pocket‐like area in the distal bile duct in five cases (5/184; 2.7%) and the authors encouraged the invention of a new device to be used for such small stones.

Based on these previous reports, the selection of the optimal stone extraction device according to the diameter of the bile duct and the size and number of stones is an important issue. Furthermore, from the standpoint of cost‐effectiveness, it is desirable to complete stone extraction with a single device as much as possible. However, few studies focusing on a specific device have been reported.

The new eight‐wire basket catheter we used in this study is made of nitinol, which is flexible and easily adapts to the shape of bile ducts and stones than conventional stainless‐steel catheters. In addition, the wire portion at the tip can be rotated by operating the handle, facilitating the capture of stones. Therefore, the characteristic spherical structure can be maintained even in narrow bile duct lumens, and the device is expected to improve the success rate of small stone extraction. The shape of the basket was maintained along the lumen in a 10‐mm diameter test tube, and a 5‐mm diameter bead ball was easily grasped in a mock‐up experiment. Additionally, complete stone extraction was achieved in 86.1% of included patients using the new eight‐wire catheter alone without the use of a balloon catheter. This success rate seems to be satisfactory for such challenging small bile duct stones and comparable to or even better than what has been reported in previous related studies.^5^, ^6^, ^15^ This encourages and supports the use of the new catheter for the endoscopic extraction of small bile duct stones even from the start as a first‐line for management.

Multivariate analysis showed that the maximum bile duct diameter and stone diameter were independent risk factors for difficulty in stone extraction with a single device. This indicates that the eight‐wire catheter is particularly effective for small stone extraction. The additional use of the balloon catheter was relatively frequent in cases of stones ≥6 mm. Although the addition of a balloon was used in many cases of stones ≥6 mm, the reason may be that the eight‐wire structure of the basket made it slightly more difficult to hold stones in proportion to the diameter of the stones. Additionally, in cases where the diameter of the bile duct was large, it is generally more difficult for all basket catheters, not only this catheter, to capture small floating stones compared with balloon catheters. The bile duct diameter tended to be slightly larger in the group with stone diameters ≥6 mm, which may be a reason for the relatively lower success rate of stone extraction with the basket catheter alone.

Although there is no study, to the best of our knowledge, regarding the clinical outcomes of conventional eight‐wire basket catheters, Ishiwatari et al. reported that basket catheters (eight‐wire in the tip and four‐wire in the proximal structure) were significantly inferior to balloon catheters in terms of the success rate of stone extraction in cases of multiple stones (four or more). The number of stones was not a risk factor for difficulty in stone extraction in our study, which may be an advantage of the nitinol structure of this catheter.

The success of endoscopic treatment of common bile duct stones depends not only on device selection, but also on the choice of appropriate techniques such as EST or EPBD, the experience of the surgeon, and the general condition of the patient, and the anatomic characteristics of the papilla.^4^, ^12^, ^16^, ^17^, ^18^ In this study, the patient's general condition, the experience of the surgeon, the presence or absence of cholangitis, diverticulitis, or trainee procedures were not the risk factors for difficulty in stone removal. There are several limitations in this study, including the fact that it is a retrospective and noncomparative study performed by many endoscopists with variable degrees of experience; no fixed rules for time to be consumed, the number of trials, type or dilution of used contrast, use of EST and/or EPBD; and the exclusion of cases where multiple basket catheters were used. Moreover, we did not routinely prospectively investigate for the recurrence of the stones when there were no symptoms.

In conclusion, the clinical outcome of the new eight‐wire basket catheter for common bile duct stones >10 mm in diameter was acceptable. It was observed that the device is particularly effective for stones <6 mm. A prospective multicenter comparative study with a larger number of patients should be conducted for further evaluation of the new eight‐wire basket.

## CONFLICT OF INTEREST

The authors declare no conflict of interest.

## FUNDING INFORMATION

All authors disclosed no financial relationships relevant to this study.

## Supporting information


**Table S1** Comparison between the success and failure groups in stone extraction with the eight‐wire catheter alone per each risk factor.
