# Supplementary material for: Application and usefulness of a new eight‐wire basket catheter for endoscopic extraction of small common bile duct stones: A retrospective multicenter study
Source: DEN Open. 2022 Jun 5;3(1):e138. doi: 10.1002/deo2.138 (PMC9307736; doi:10.1002/deo2.138)
Supplement: Supplementary file 1 — Table S1 Comparison between the success and failure groups in stone extraction with the eight‐wire catheter alone per each risk factor. [file DEO2-3-e138-s001.docx]

Supplementary Table 1. Comparison between the success and failure groups in stone extraction with the eight-wire catheter alone per each risk factors.

| Parameters | Success group  (n = 124) | Failed group  (n = 20) |
| --- | --- | --- |
| Sex, Male, n (%), | 78 (62.9) | 11 (55.0) |
| Age, year (mean ± SD) | 72.8 ± 13.7 | 76.2 ± 7.6 |
| Duodenum diverticulum, n (%) | 34 (27.4) | 6 (30.0) |
| Acute cholangitis before ERCP, n (%) | 36 (29.0) | 5 (25.0) |
| Trainee, n (%) | 51 (41.1) | 7 (35.0) |
| EST, n (%) | 101 (81.4) | 14 (70.0) |
| EPBD, n (%) | 18 (14.5) | 5 (25.0) |
| Number of stones, n (mean ± SD) | 2.4 ± 6.7 | 2.7 ± 4.3 |
| Diameter of the largest stone, mm (mean ± SD) | 4.9 ± 4.3 | 6.5 ± 5.7 |
| Maximum diameter of common bile duct, mm (mean ± SD) | 10.1 ± 12.8 | 12.6 ± 5.7 |

ERCP: endoscopic retrograde cholangiopancreatography, EST: endoscopic sphincterotomy, EPBD: endoscopic papillary balloon dilation
